# Solid-Phase Electrochemiluminescence Enzyme Electrodes Based on Nanocage Arrays for Highly Sensitive Detection of Cholesterol

**DOI:** 10.3390/bios14080403

**Published:** 2024-08-21

**Authors:** Xinying Ma, Zhe Zhang, Yanyan Zheng, Jiyang Liu

**Affiliations:** School of Chemistry and Chemical Engineering, Zhejiang Sci-Tech University, Hangzhou 310018, China; 2023221002036@mails.zstu.edu.cn (X.M.); 2023211001069@mails.zstu.edu.cn (Z.Z.); 201920103007@mails.zstu.edu.cn (Y.Z.)

**Keywords:** solid-phase electrochemiluminescence, enzyme electrode, nanocage array, vertically aligned mesoporous silica film, metabolite

## Abstract

The convenient and sensitive detection of metabolites is of great significance for understanding human health status and drug development. Solid-phase electrochemiluminescence (ECL) enzyme electrodes show great potential in metabolite detection based on the enzyme-catalyzed reaction product hydrogen peroxide (H_2_O_2_). Herein, a solid-phase ECL enzyme sensor was fabricated based on a confined emitter and an immobilized enzyme using electrostatic nanocage array, constructing a platform for the sensitive detection of cholesterol. The electrostatic cage nanochannel consists of a bipolar and bilayer vertically aligned mesoporous silica film (bp-VMSF). The upper layer of bp-VMSF is an amino-modified, positively charged VMSF (p-VMSF), and the lower layer is a negatively charged VMSF (n-VMSF). The most commonly used ECL probe tris(bipyridine)ruthenium(II) (Ru(bpy)_3_^2+^) is fixed in n-VMSF by electrostatic adsorption from n-VMSF and electrostatic repulsion from the upper p-VMSF, generating significantly enhanced and stable ECL signals. The successful preparation of the electrostatic cage was characterized by scanning electron microscopy (SEM) and electrochemical methods. After amino groups on the outer surface of bp-VMSF were derivatized with aldehyde, cholesterol oxidase (ChOx) molecules were covalently immobilized. The successful construction of the enzyme electrode was characterized by cyclic voltammetry (CV) and electrochemical impedance spectroscopy (EIS). When the corresponding enzyme substrate, cholesterol, was present in the solution, the ECL signal of Ru(bpy)_3_^2+^ was quenched by the enzyme-catalyzed reaction product H_2_O_2_, enabling the high-sensitivity detection of cholesterol. The linear range for detecting cholesterol was from 0.05 mM to 5.0 mM, with a limit of detection (LOD) of 1.5 μM.

## 1. Introduction

Metabolite detection is crucial for understanding human health status, disease risks, and responses to the environment and drugs owing to the detailed information about the chemical processes within an organism provided by metabolites [1,2,3]. In addition, metabolite detection can be employed for biomarker or drug discovery and medical diagnosis, facilitating personalized medicine and drug therapy [4,5,6,7]. For instance, cholesterol, as a lipid, is vital for normal bodily functions; however, elevated levels may increase the risk of cardiovascular diseases [8]. By regularly monitoring cholesterol levels, high cholesterol can be detected and controlled in a timely manner, thereby reducing the risk of cardiovascular diseases [9]. Therefore, the development of convenient and rapid cholesterol detection platforms is of great significance.

Enzymes are highly efficient catalysts in nature, widely employed in metabolite detection [10]. For example, commonly used enzymes in cholesterol detection include cholesterol esterase and cholesterol oxidase, which catalyze the hydrolysis and oxidation reactions of cholesterol, generating measurable signals with which to determine cholesterol concentrations [11]. However, enzymes suffer from issues of long-term operational instability and complex recycling, limiting their applications in the field of biosensing. Immobilized enzymes reduce enzyme dosage and may enable reuse, while offering high stability. Thus, immobilized, oxidase-based biosensors, relying on the detection of H_2_O_2_ as a product of enzyme-catalyzed reactions, have been utilized for the detection of cholesterol [12,13,14]. In recent years, electrochemiluminescence (ECL) enzyme sensors demonstrated significant potential in metabolite analysis [12,13,14]. ECL is a technique combining electrochemistry with chemiluminescence, where the application of voltage or current to the electrode surface induces redox reactions of electroactive species, leading to the generation of excited-state species and, ultimately, luminescence [15,16,17]. ECL technology offers advantages such as low background current, fast response rate, and good controllability [18,19]. Among various ECL emitters, tris(2,2′-bipyridyl)ruthenium(II) (Ru(bpy)_3_^2+^) is widely used due to its excellent stability, water solubility, and ECL efficiency [20]. This cationic ECL emitter can also be efficiently enriched by negatively charged nanomaterials through electrostatic interactions. Co-reactants such as tripropylamine (TPA) can interact with [Ru(bpy)_3_]^2+^, resulting in a strong ECL emission. It has been reported that the ECL signal of the [Ru(bpy)_3_]^2+^/TPA system is easily quenched by the H_2_O_2_ present in the solution [21]. Thus, the ECL detection of cholesterol can be achieved by monitoring the H_2_O_2_ generated by enzyme-catalyzed reactions.

Two strategies are commonly employed in ECL detection [22,23,24,25,26]. One involves a solution-based ECL emitter, commonly known as the homogeneous ECL [27,28,29]. The other immobilizes the ECL emitter on the surface of an electrode, known as the solid-phase ECL [30]. Compared to the former, the latter holds potential for reagent less detection [31]. In recent years, nanomaterials have been widely used as electrode modification materials to enhance sensing performance [32,33,34]. The efficient immobilization of the ECL emitter on electrodes is crucial for constructing solid-phase ECL sensors with high performance. Porous materials with high surface area hold potential in electrode modification and the immobilization of functional substances [35,36,37,38,39]. Vertically aligned mesoporous silica film (VMSF) features uniform, ultrasmall nanochannels and exhibits advantages such as excellent molecular permeability and anti-contamination properties [40,41,42,43]. Due to their molecular-level charge-selective permeability, VMSF holds promise in ECL sensor construction. The deprotonation of abundant silanol groups on the inner walls of VMSF renders a VMSF negative charge, enabling the electrostatic adsorption of a positively charged ECL emitter like Ru(bpy)_3_^2+^, significantly enhancing the sensitivity of ECL sensors [44]. For instance, Su et al. immobilized the derivative of ruthenium(II) complex within nano-sized cages composed of dual-layered VMSF with different nanochannel diameters, providing insights into the fabrication of solid-state ECL sensors [45]. However, the water solubility of the selected ECL emitter was poor, and the preparation process of nano-sized cages was complex. Utilizing VMSF-based functional structures with a simple construction method holds great potential for the fabrication of solid-phase ECL systems for highly sensitive metabolite detection.

**Figure 1:**
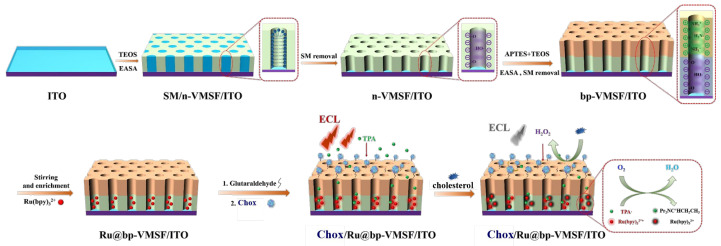
A schematic illustration of the construction of solid-state ECL sensor with immobilized ECL emitter and oxidase for sensing of small metabolic.

In this work, a solid-state ECL sensing platform was fabricated based on the bipolar VMSF with an oppositely charged surface (bp-VMSF) for sensitive detection of cholesterol. As shown in Figure 1, an inexpensive and transparent indium tin oxide (ITO) electrode was used as the supporting electrode. VMSFs with negative charges (n-VMSF) and positive charges (p-VMSF) were sequentially grown on an ITO surface using the electrochemical-assisted self-assembly (EASA) method. The efficient and stable immobilization of the ECL emitter, Ru(bpy)_3_^2+^, was achieved using the bp-VMSF, generating a stable ECL signal. Cholesterol oxidase was used as the model enzyme to be covalently immobilized on the outer surface of the bp-VMSF. The enzyme-catalyzed oxidation of the metabolite product, H_2_O_2_, which could quench the localized ECL signal of Ru(bpy)_3_^2+^, enabling the detection of cholesterol, combined with the fouling-resistant and anti-interference properties of the VMSF, allowed the content of cholesterol in the human serum samples to be determined.

## 2. Materials and Methods

### 2.1. Chemicals and Materials

Tetraethyl orthosilicate (TEOS, 98%), cetyltrimethylammonium bromide (CTAB), potassium hexacyanoferrate(III) (K_3_[Fe(CN)_6_], 99.5%), potassium hexacyanoferrate(II) (K_4_[Fe(CN)_6_], 99.5%), bovine serum albumin (BSA), 3-aminopropyltriethoxysiloxane (APTES), glucose (Glu), cholesterol (ChO), cholesterol oxidase (ChOx), fetal bovine serum, lactose, maltose, fructose, ascorbic acid (AA), dopamine (DA), or L-cysteine (L-cys), potassium hydrogen phthalate (KHP) and glutaraldehyde (GA) were all purchased from Aladdin Biochemical Technology Co., Ltd. (Shanghai, China). Tris(bipyridine)ruthenium(II) chloride hexahydrate (Ru(bpy)_3_Cl_2_·6H_2_O) was purchased from Sigma-Aldrich (Shanghai, China). Sodium nitrate (NaNO_3_) was obtained from Xinsong Chemical Reagent Co., Ltd. (Wuxi, China). Ethanol (99.8%) and sodium hydroxide were purchased from Gaojing Fine Chemical Co., Ltd. (Hangzhou, China). Phosphate buffer solution was prepared by mixing Na_2_HPO_4_ and NaH_2_PO_4_ in certain proportions. All chemicals were of analytical grade and used as received. Solutions were prepared using ultrapure water (18.2 MΩ·cm). Indium tin oxide (ITO) conductive glass (sheet resistance < 17 Ω/sq, ITO thickness: 100 ± 20 nm) was purchased from Kewei Optoelectronics Technology Co., Ltd. (Zhuhai, China). Before use, ITO was sonicated in 1 M NaOH for 1 h, followed by sonication in acetone, ethanol, and ultrapure water for 0.5 h each, and then drying under nitrogen gas. 

### 2.2. Measurements and Instrumentations

The thickness of bp-VMSF was characterized using a scanning electron microscope (SEM, Hitachi SU8010, Tokyo, Japan) at an accelerating voltage of 5 kV. To prepare the SEM sample, a scratch was made on the electrode surface using a glass cutter, and a fresh cross-section was obtained. After gold sputtering, the sample was observed using SEM. Electrochemical impedance spectroscopy (EIS), cyclic voltammetry (CV), and differential pulse voltammetry (DPV) tests were performed on an Autolab (PGSTAT302N) electrochemical workstation (Metrohm, Zurich, Switzerland). The frequency range for EIS measurement was 0.1–1 × 10^5^ Hz. The parameters for DPV measurement, including step potential, pulse amplitude, pulse time, and interval time, were 0.005 V, 0.05 V, 0.05 s, and 0.2 s, respectively.

### 2.3. Preparation of bp-VMSF-Modified Electrode

The electrostatic nanocage in bp-VMSF is composed of two-layered VMSF with asymmetrical surface-charges. The upper layer consists of positively charged amine-modified VMSF (p-VMSF), while the lower layer comprises negatively charged nanochannels (n-VMSF). The supporting ITO electrode was immersed in an acidic precursor solution containing siloxane and a cationic surfactant (cetyltrimethylammonium bromide, CTAB) and then a constant current under negative potential was applied on ITO. At this time, the cathodic reduction of water occurred on the electrode surface, generating a large amount of OH^-^ ions, causing a local pH increase [46], inducing the condensation reaction of siloxane and self-assembly of surfactant micelles (SM), and ultimately resulting in vertically aligned mesoporous silica nanochannels with CTAB templates. The EASA method enables the rapid growth of VMSF. Multiple layers of VMSF films can also be prepared using the EASA method [46,47,48,49,50,51]. By adding amino-functionalized siloxane reagent (APTES), amino-functionalized p-VMSF can be simply prepared. The template SM in the nanochannels can be conveniently removed by immersing the electrode in an HCl–ethanol solution. Specifically, the n-VMSF was grown on cleaned ITO glass using the EASA method [46]. Specifically, 1.585 g of CTAB and 2.833 g of TEOS were added to a mixture solution of 20 mL ethanol and 20 mL of 0.1 M NaNO_3_ (pH 2.6). The obtained solution was stirred at room temperature for 2.5 h to obtain the precursor solution. Then, n-VMSF was grown for 10 s when constant current (current density of -1.3 mA/cm^2^) was employed on ITO. The obtained electrode was quickly removed and thoroughly washed with ultrapure water. Then, the electrode was aged overnight at 120 °C to obtain an electrode containing micelles (SM) in the nanochannels (SM@VMSF/ITO). The SM@VMSF/ITO electrode was stirred in a 0.1 M HCl–ethanol solution for 5 min to remove the micelles, resulting in a single-layer VMSF-modified electrode with an open nanochannel (n-VMSF/ITO). The n-VMSF/ITO electrode was further grown with p-VMSF in a precursor solution containing APTES. Specifically, 1 mM APTES was added to the precursor solution, and the pH was adjusted to 3 with concentrated HCl. The solution was stirred at room temperature for 2.5 h to obtain a precursor solution containing amino-functionalized siloxane. After the same EASA growth, washing, and aging steps, a double-layer VMSF-modified electrode containing SM was obtained (SM@bp-VMSF/ITO). After SM was removed by stirring in a 0.1 M HCl–ethanol solution for 5 min, an ITO electrode modified with bp-VMSF was obtained (bp-VMSF/ITO).

### 2.4. Preparation of Solid-Phase ECL Enzyme-Sensing Platform

Glutaraldehyde (GA) was then utilized as a bifunctional cross-linker for the fabrication of enzyme-immobilized electrodes, which react with the amino groups on bp-VMSF and the amino acid residues on the cholesterol oxidase molecules (ChOx) through a Schiff base reaction. Specifically, a bp-VMSF/ITO electrode was immersed in a 5% GA solution and allowed to react in the dark for 30 min. Afterward, unbound GA was removed with 0.01 M PBS to obtain an aldehyde-functionalized electrode (GA/bp-VMSF/ITO). The ECL emitter Ru(bpy)_3_^2+^ was then electrostatically immobilized in n-VMSF and effectively repelled by the upper p-VMSF, thereby being confined within the electrostatic nanocage. Specifically, the GA/bp-VMSF electrode was then incubated in a 10 mM Ru(bpy)_3_^2+^ solution with stirring for 1 h to enrich Ru(bpy)_3_^2+^. After unbound Ru(bpy)_3_^2+^ was thoroughly washed with 0.01 M PBS, the electrode with immobilized Ru(bpy)_3_^2+^ was obtained (Ru@GA/bp-VMSF/ITO). The Ru@GA/bp-VMSF electrode was then separately incubated overnight at 4 °C with a solution of cholesterol oxidase. After washing away the unbound oxidase with 0.01 M PBS, the solid-phase ECL sensing platform with an immobilized ECL probe and enzyme (ChOx/Ru@GA/bp-VMSF) was obtained. The sensors were then immersed in solutions containing different concentrations of cholesterol, and the ECL signals were measured for the detection of cholesterol levels.

### 2.5. ECL Detection of Cholesterol

With enzymes immobilized on the electrode surface, the enzyme-catalyzed reaction product H_2_O_2_, in the presence of the corresponding cholesterol, can rapidly diffuse from the interface to the underlying electrode. Based on the ECL signal of Ru(bpy)_3_^2+^/TPA quenched by H_2_O_2_, the highly sensitive detection of cholesterol could be achieved. Briefly, in a 0.01 M PBS (pH = 7.4) solution containing 10 μM Ru(bpy)_3_^2+^ and 3 mM TPA, different concentrations of cholesterol were added, and the ECL signals of the constructed solid-phase ECL enzyme electrode were measured. For ECL measurement, the applied CV scan rate was 100 mV/s with a potential range from 0 to 1.25 V. The voltage of the photomultiplier tube was 400 V. Real sample analysis was performed, using the standard addition method to detect cholesterol in fetal bovine serum. The serum was diluted 50 times with PBS (0.01 M, pH = 7.4) buffer solution before direct measurement.

## 3. Results and Discussion

### 3.1. Characterization of bp-VMSF

**Figure 2:**
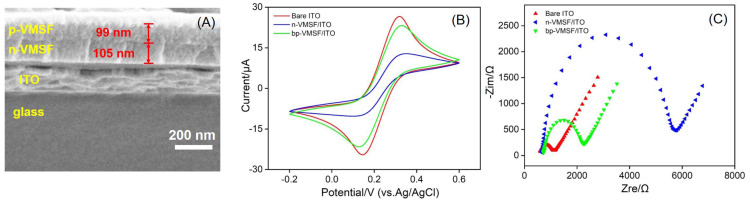
(**A**) SEM image of cross-section of bp-VMSF/ITO. (**B**) CV and (**C**) EIS curves of bare ITO, n-VMSF/ITO and bp-VMSF/ITO electrodes in 0.05 M KHP (pH = 4) containing 0.5 mM K_3_[Fe(CN)_6_]. Scan rate in (**B**) is 50 mV/s.

As shown in Figure 1, a new ECL sensing platform was fabricated with the immobilized ECL emitter and cholesterol oxidase using an electrostatic nanocage array of bp-VMSF. The morphology of bp-VMSF was characterized using SEM. As shown in Figure 2A, the bp-VMSF/ITO electrode consists of layers from top to bottom, including the p-VMSF layer, n-VMSF layer, ITO layer, and glass substrate. The thickness of the p-VMSF layer is 99 nm, and the thickness of the n-VMSF layer is 105 nm. The properties of bp-VMSF/ITO were further investigated using cyclic voltammetry (CV) and electrochemical impedance spectroscopy (EIS). As depicted in Figure 2B, the negatively charged Fe(CN)_6_^3−^ exhibited a distinct oxidation/reduction peak on bare ITO. However, the peak current of Fe(CN)_6_^3−^ measured on the n-VMSF/ITO electrode decreased, attributable to the electrostatic repulsion between Fe(CN)_6_^3−^ and the negatively charged n-VMSF. Upon further growth of the positively charged p-VMSF on the n-VMSF/ITO electrode, the current of Fe(CN)_6_^3−^ on bp-VMSF/ITO increased, indicating the adsorption effect of p-VMSF on the negatively charged probe. In the EIS spectra (Figure 2C), each curve comprises a semicircle in the high-frequency region and a linear segment in the low-frequency region. The semicircle represents processes limited by electron transfer, while the linear portion signifies diffusion-limited processes. It is evident that upon modifying ITO with n-VMSF, which electrostatically repulses Fe(CN)_6_^3−^, there is a notable increase (2684.9 Ω) in the apparent charge transfer resistance (*R*_et_) compared to that of the unmodified ITO (280.8 Ω). Subsequent modification with p-VMSF results in a decrease in *R*_et_ (744.6 Ω), suggesting electrostatic attraction towards Fe(CN)_6_^3−^. These results are consistent with those obtained in the CV measurement.

### 3.2. Stability of Ru(bpy)_3_^2+^ Fixed in bp-VMSF

**Figure 3:**
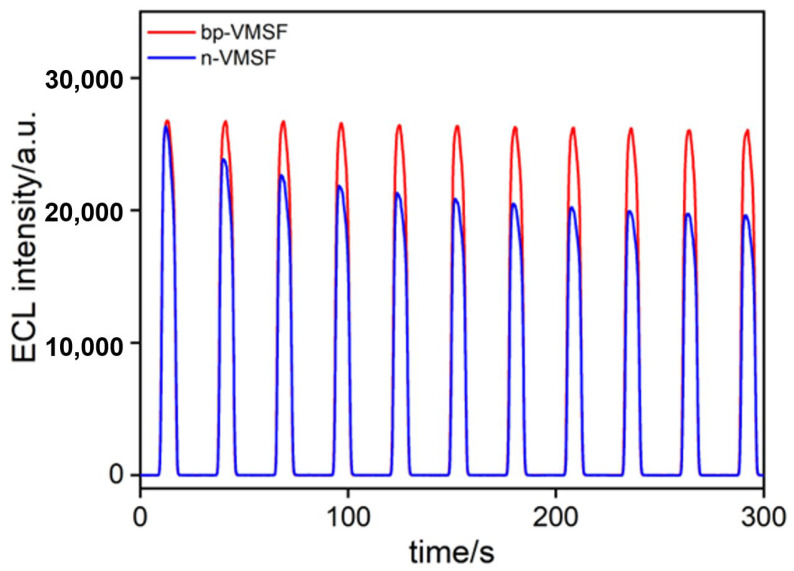
The ECL signal obtained under continuous scanning when Ru(bpy)_3_^2+^ was fixed in bp-VMSF or n-VMSF.

In this study, Ru(bpy)_3_^2+^ is confined electrostatically within the nanochannels of bp-VMSF/ITO electrodes and serves as an immobilized probe generating ECL signals. Therefore, its stability significantly influences the performance of the fabricated ECL sensors. The stability of the ECL signals on electrodes fixed with Ru(bpy)_3_^2+^ in bp-VMSF (Ru@bp-VMSF/ITO) and n-VMSF (Ru@n-VMSF/ITO) was compared, as shown in Figure 3. It can be observed that the ECL signal of the Ru@n-VMSF/ITO electrode gradually weakens with increasing scan time, indicating the gradual leakage of Ru(bpy)_3_^2+^ during continuous scanning. Thus, the ECL emitter cannot be stably fixed in the n-VMSF as the Ru(bpy)_3_^2+^ gradually detaches from the electrode surface via concentration diffusion. In contrast, the ECL signal of the Ru@bp-VMSF/ITO remains unchanged from its initial value even after 300 s of measurement, indicating the high stability of the fixed Ru(bpy)_3_^2+^. This good stability can be ascribed to the dual electrostatic forces from the bp-VMSF acting on Ru(bpy)_3_^2+^, including the electrostatic attraction of the inner layer n-VMSF and the electrostatic repulsion of the outer layer p-VMSF. This dual electrostatic force enabled the Ru(bpy)_3_^2+^ to remain stable within the bp-VMSF channels. Thus, utilizing bp-VMSF to immobilize the ECL emitter eliminates the need for complex preparation processes and reduces the usage of Ru(bpy)_3_^2+^, providing a convenient strategy for constructing solid-state ECL sensing platforms. Thanks to the significant enrichment of Ru(bpy)_3_^2+^ by the bp-VMSF, the concentration of Ru(bpy)_3_^2+^ used is much lower than that in conventional homogeneous ECL systems (typically 0.1–0.2 mM), which will greatly reduce the detection cost of the sensor.

### 3.3. Feasibility for the Fabrication of Enzyme-Immobilized Electrodes

**Figure 4:**
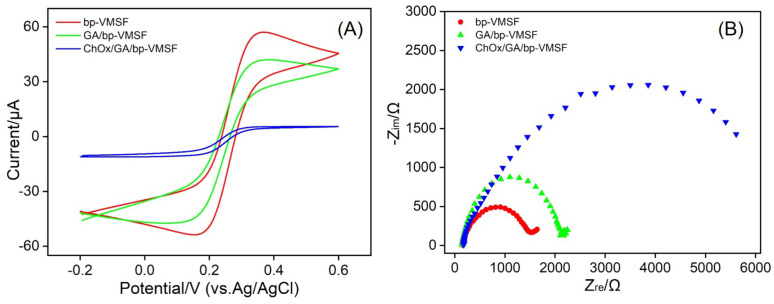
(**A**) CV curves obtained on different electrodes in 0.1 M KCl solution containing 2.5 mM Fe(CN)_6_^3−/4−^. Scan rate was 50 mV/s. (**B**) EIS plots obtained on different electrodes in 0.1 M KCl solution containing 2.5 mM Fe(CN)_6_^3−/4−^.

The electrochemical signals of different electrodes obtained during the preparation of enzyme-immobilized electrodes were investigated using electrochemical methods. Figure 4A shows the CV response of Fe(CN)_6_^3−/4−^ on electrodes modified with bp-VMSF, GA/bp-VMSF, or ChOx/GA/bp-VMSF. Compared to the bp-VMSF/ITO electrode, the oxidation–reduction current of Fe(CN)_6_^3−/4−^ measured on the aldehyde-derived GA/bp-VMSF/ITO electrode slightly decreased, possibly due to the changing of the surface through cross-linking of amine groups by GA. Upon further covalent immobilization of cholesterol oxidase, the oxidation–reduction peak current of Fe(CN)_6_^3−/4−^ measured on the ChOx/GA/bp-VMSF/ITO electrode further decreased. This is attributed to the non-conductivity and spatial hindrance of the enzyme, which affects the diffusion of Fe(CN)_6_^3−/4−^ probes to the supporting electrode, resulting in a decrease in the probe signal. This result demonstrates the successful immobilization of ChOx on bp-VMSF. Figure 4B depicts the EIS curves obtained on different electrodes. Compared to that of bp-VMSF/ITO (724.2 Ω), the *R*et of the electrode modified with GA slightly increased (1043.9 Ω). Furthermore, after further modification with cholesterol oxidase, the large spatial hindrance of the enzyme and its non-conductive properties inhibited the charge transfer between Fe(CN)_6_^3−/4−^ and the electrode, leading to a significantly increased *R*et (3498.5 Ω). All these results indicate the successful preparation of ChOx/GA/bp-VMSF electrodes.

### 3.4. Feasibility of ECL Detection of Cholesterol Using Enzyme-Immobilized Electrode

**Figure 5:**
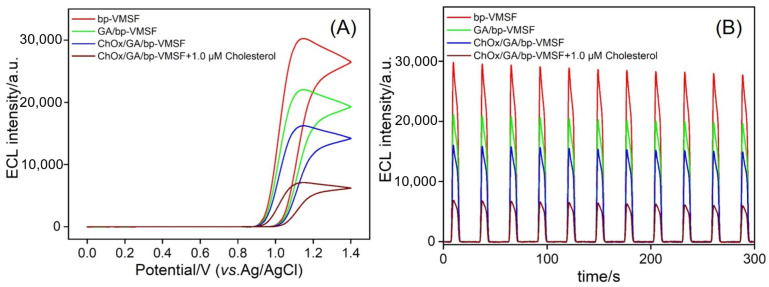
ECL intensity obtained on different electrodes under different potential (**A**) or scan time (**B**) in 10 μM Ru(bpy)_3_^2+^ and 3 mM TPA in 0.01 M PBS (pH = 7.4). Scan rate in (**A**) was 100 mV/s.

The ECL response during the sensor preparation process was further investigated. Electrodes obtained from each modification step were immersed in a buffer solution containing Ru(bpy)_3_^2+^ and co-reactant TPA to obtain ECL response curves. As shown in Figure 5A,B, after GA modification, the ECL signal decreased. When cholesterol oxidase was immobilized on the electrode, the ECL signal further decreased due to the enzyme’s large spatial hindrance, which reduced the diffusion of the co-reactant TPA and electrolyte into the nanochannels to react with the ECL emitter Ru(bpy)_3_^2+^. This is consistent with the results of the CV and EIS. In the presence of cholesterol in the solution, the enzymatic reaction between cholesterol oxidase and cholesterol generates hydrogen peroxide, which effectively quenches the ECL signal of the Ru(bpy)_3_^2+^/TPA system. Electrodes obtained from each modification step were continuously scanned in the solution for 300 s, and the relative standard deviation (RSD) value of the ECL signal was within 5%, indicating that the prepared sensor had good stability. By simply replacing the immobilized enzyme, the prepared sensor can be expanded into a universal platform for detecting other metabolites.

In the Ru(bpy)_3_^2+^/TPA system, the ECL emitter Ru(bpy)_3_^2+^ on the electrode surface is oxidized to the strong oxidant Ru(bpy)_3_^3+^, while TPA is oxidized to form the cationic radical TPA^•+^. The TPA^•+^ rapidly loses a proton to form the strongly reducible TPA^•^, and Ru(bpy)_3_^3+^ reacts with TPA^•^ to generate the excited state Ru(bpy)_3_^2+*^, which returns to the ground state and emits light. Furthermore, Ru(bpy)_3_^2+^ is regenerated after emitting light, making Ru(bpy)_3_^2+^ a reusable emitter. The mechanism is shown as Equations (1)–(5):

Ru(bpy)_3_^2+^ − e^−^ → Ru(bpy)_3_^3+^ (1)

Ru(bpy)_3_^3+^ + TPA → Ru(bpy)_3_^2+^ + TPA^•+^ (2)

TPA^•+^ → TPA^•^ + H^+^ (3)

Ru(bpy)_3_^3+^ + TPA^•^ → Ru(bpy)_3_^2+*^+ products (4)

Ru(bpy)_3_^2+*^ → Ru(bpy)_3_^2+^ + hν (5)

The quenched ECL in the presence of exogenous hydrogen peroxide was investigated. Figure S1 in the supporting information (SI) displays the ECL signals obtained on the bp-VMSF-modified electrode under different potentials (Figure S1A) or scan times (Figure S1B) in the absence or presence of H_2_O_2_ in Ru(bpy)_3_^2+^/TPA system. As seen, the added H_2_O_2_ quenched the ECL of the Ru(bpy)_3_^2+^/TPA system. When H_2_O_2_ is present in the solution, Ru(bpy)_3_^3+^ and TPA^•^ in the above ECL mechanism are easily quenched by H_2_O_2_ or the free radicals generated by H_2_O_2_ or H_2_O_2_ oxidation, leading to a decrease in ECL intensity [45,52]. Additionally, the decomposition of H_2_O_2_ produces O_2_, which acts as a quencher for Ru(bpy)_3_^2+*^, thus achieving a dual quenching effect. The quenching reactions are as follows:

TPA^•^ + O_2_ → Pr_2_NC^+^HCH_2_CH_3_ + P2 (6)

Here, P2 represents the reduction product of O_2_. This reaction competes with the ECL pathway by consuming intermediate species, thereby reducing the ECL intensity.

### 3.5. Performance for ECL Detection of Cholesterol

**Figure 6:**
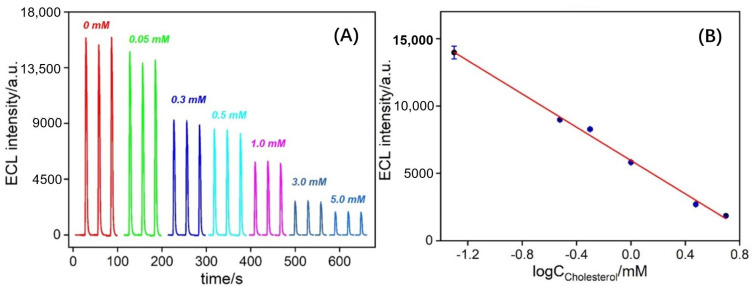
(**A**) ECL response obtained on ChOx/GA/bp-VMSF electrode towards different concentrations of cholesterol. (**B**) Linear calibration curve for cholesterol detection. Error bar represents the standard deviation of three measurements.

The prepared solid ECL sensors were utilized to detect a series of different concentrations of cholesterol. Figure 6 depicts the ECL signals obtained from ChOx/GA/bp-VMSF/ITO electrodes after incubation with various concentrations of cholesterol, respectively. As shown in Figure 6A, the intensity of ECL decreases with increasing cholesterol concentration. This is attributed to the higher quenching effect of H_2_O_2_, the product of the enzymatic reaction catalyzed by cholesterol oxidase, on the ECL signal of the Ru(bpy)_3_^2+^/TPA system with increasing cholesterol concentration. A linear relationship between the ECL signal (*I*_ECL_) and the logarithm of cholesterol concentration (log*C*_cholesterol_) is observed (Figure 6B), with a detection linear range from 0.05 mM to 5.0 mM (*I*_ECL_ = −6175.2 log*C*_cholesterol_ + 5953.2, *R*^2^ = 0.995). The limit of detection (LOD), calculated based on the signal-to-noise ratio (S/N), is determined to be 1.5 μM. A comparison between the determination of cholesterol using different electrodes is demonstrated in Table S1 (SI) [53,54,55,56,57,58,59]. The LOD is only higher than that obtained from the ECL detection using a glassy carbon electrode (GCE) modified with graphitic carbon nitride and black phosphorus nanosheets (g-C_3_N_4_/BPNSs/GCE) [56]. The starting concentration in the detection linear range is higher than that obtained using an electrochemical (EC) detection platform constructed via the modification of fluorine tin oxide (FTO) with β-cyclodextrin/benzoquinone/cysteamine/nitrogen-doped graphene quantum dots/(3-aminopropyl) triethoxysiloxane (β-CD/COOH/BQ/Cys/N-GQDs/APTES/FTO) [53], or using carbon paste electrode modified with microorganism–catalase/ionic liquid/multiwalled carbon nanotubes and (MO-Catalase/IL/MWCNT/CPE) [55], or based on gold nanoparticles and single-stranded DNA-reduced graphene oxide nanocomposites based electrochemical biosensor (ChOx/AuNPs/ssDNA-rGO/GCE) [59], or ECL detection based on g-C_3_N_4_/BPNSs/GCE [56], or GCE modified with carbonized polydopamine nanotubes and cholesterol oxidase (C-PDANTs@ChOx-GCE) [56]. But the maximum concentration in the detection linear range is only lower than the ECL detection when using 3D-printed closed bipolar ECL devices [54] or EC detection based on screen-printed electrodes modified with nanoporous gold (NPG/SPE) [58].

### 3.6. Selectivity of the Fabricated Sensors

**Figure 7:**
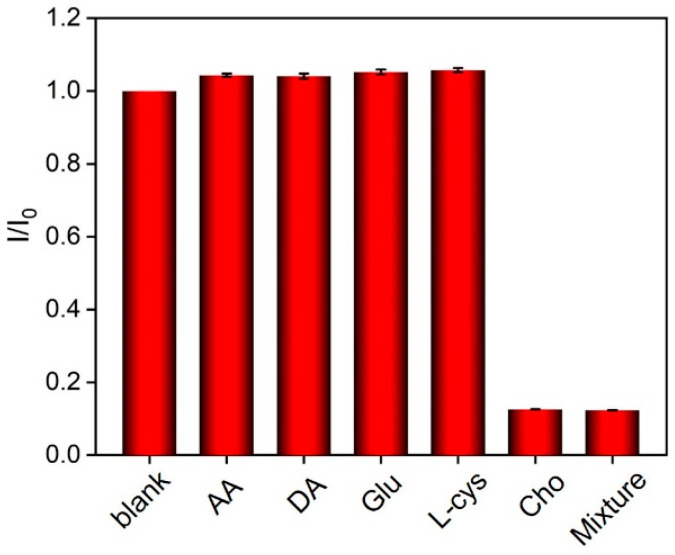
The ratio of ECL intensity (I/I_0_) obtained on ChOx/GA/bp-VMSFin absence (I_0_) or presence (I) of possible interfering species (150 mM), glucose (Glu, 3 mM) or cholesterol (Cho, 3 mM), or their mixture.

To further explore the detection selectivity of the enzyme-immobilized electrodes, the prepared electrodes were employed to detect a series of biomolecules or their mixtures. As shown in Figure 7, for the ChOx/GA/bp-VMSF/ITO electrode, the ECL signal remained unchanged when other biomolecules, such as lactose, maltose, fructose, or the macromolecule bovine serum albumin (BSA), were present in the solution, even at 50-fold concentrations. Significant decreases in ECL signals were observed only when cholesterol or mixtures containing cholesterol were present in the solution, even in the presence of other biomolecules such as ascorbic acid (AA), dopamine (DA), glucose (Glu), or L-cysteine (L-cys) at 50-fold concentrations. Thus, only when the immobilized enzyme electrode was incubated with its substrate did the ECL signal significantly decrease, demonstrating the high selectivity of the constructed enzyme electrodes for detecting target metabolites. 

### 3.7. Real Sample Analysis

The application of the constructed sensors in real sample analysis was evaluated using a standard addition method. As shown in Table 1, the ChOx/GA/bp-VMSF/ITO electrode exhibited good recovery (99.3% to 110%) and small RSD values (<2.1%) for the detection of cholesterol in fetal bovine serum diluted 50 times. This indicates that the detection using the fabricated sensors has good accuracy for detecting cholesterol in real samples.

## 4. Conclusions

In summary, a solid-phase ECL enzyme-sensing platform was fabricated for the highly sensitive detection of cholesterol. Two layers of oppositely charged VMSF (bp-VMSF) were rapidly grown on ITO electrodes using the EASA method. By utilizing the electrostatic attraction of the inner-layer VMSF towards the ECL emitter Ru(bpy)_3_^2+^ and the electrostatic repulsion of the outer-layer VMSF to the Ru(bpy)_3_^2+^, the ECL emitter was “trapped” within the nanochannels, resulting in significantly enhanced and stable ECL signals. Further, aldehyde derivatization of the amino groups on the outer layer of bp-VMSF, followed by covalent immobilization of cholesterol oxidase, resulted in the fabrication of the immobilized enzyme electrode. The enzyme-catalyzed reactions of the oxidases with cholesterol produced H_2_O_2_, effectively quenching the ECL signal of the Ru(bpy)_3_^2+^/TPA system, enabling the highly sensitive detection of cholesterol ranged from 0.05 mM to 5.0 mM with a low LOD (1.5 μM). Compared to the homogeneous ECL sensors (typically 0.1–0.2 mM of Ru(bpy)_3_^2+^), the solid-phase ECL sensor in this study greatly reduces the consumption of expensive ECL reagents (10 μM of Ru(bpy)_3_^2+^) while achieving signal amplification. Additionally, compared to homogeneous enzyme detection systems, immobilized enzymes not only decrease the enzyme dosage but also enhance detection sensitivity. Cholesterol oxidase was chosen as a model, successfully achieving the highly sensitive and selective detection of cholesterol. This study offers a convenient strategy for developing a sensitive metabolite sensing platform by changing the immobilized enzyme.

## Figures and Tables

**Table 1:** Determination of cholesterol using fabricated sensor in diluted fetal bovine serum.

Analyte	Added (mM)	Detected (mM)	RSD (%, n = 3)	Recovery (%)
cholesterol	0.50	0.55	2.1	110.0
	1.00	1.09	1.2	109.0
	4.50	4.47	0.2	99.3

## Data Availability

The data presented in this study are available on request from the corresponding author.

## Supplementary Materials

The following supporting information can be downloaded at: https://www.mdpi.com/article/10.3390/bios14080403/s1, Figure S1: ECL intensity obtained on bp-VMSF modified electrode under different potential (A) or scan time (B) in absence or presence of H_2_O_2_ in 10 μM Ru(bpy)_3_^2+^ and 3 mM TPA; Table S1: Comparison between electrochemical or electrochemiluminescence detection of cholesterol using different modified electrode.
