# Supplementary material for: Solid-Phase Electrochemiluminescence Enzyme Electrodes Based on Nanocage Arrays for Highly Sensitive Detection of Cholesterol
Source: Biosensors (Basel). 2024 Aug 21;14(8):403. doi: 10.3390/bios14080403 (PMC11353124; doi:10.3390/bios14080403)
Supplement: Supplementary file 1 [file biosensors-14-00403-s001.zip › biosensors-3132444-supplementary.pdf]

biosensors

Supporting Materials

Solid-Phase Electrochemiluminescence Enzyme Electrodes Based on Nanocage Arrays for Highly Sensitive Detection of Cholesterol

Xinying Ma †, Zhe Zhang †, Yanyan Zheng and Jiyang Liu *

School of Chemistry and Chemical Engineering, Zhejiang Sci-Tech University, Hangzhou 310018, China; 2023221002036@mails.zstu.edu.cn (X.M.); 2023211001069@mails.zstu.edu.cn (Z.Z.); 201920103007@mails.zstu.edu.cn (Y.Z.)
* Correspondence: liujy@zstu.edu.cn
† These authors contributed equally to this work.

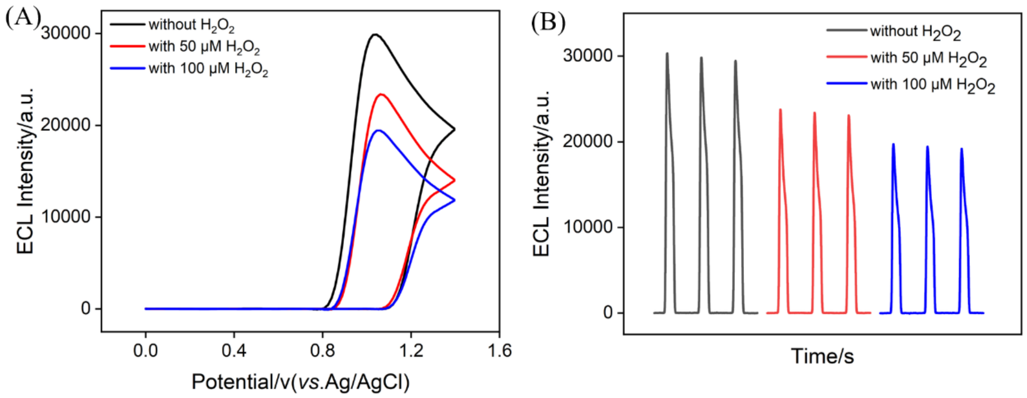

Figure S1. ECL intensity obtained on bp-VMSF modified electrode under different potential (A) or scan time (B) in absence or presence of H_2O_2 in 10 μM $Ru(bpy)_3^{2+}$ and 3 mM TPA.

Table S1. Comparison between electrochemical or electrochemiluminescence detection of cholesterol using different modified electrode. .

Scheme .	Method	Linear Range (μM)	LOD (μM)	Ref.
β-CD/COOH/BQ/Cys/N-GQDs/APTES/FTO	EC	10~50<br>60-100	2.10	53
3-D-printed closed bipolar ECL devices	ECL	500~10000	490	54
MO-Catalase/IL/MWCNT/CPE	EC	5~600	1.52	55
g-C_3N_4/BPNSs/GCE	ECL	0.5~500	0.14	56
C-PDANTs@ChOx-GCE	ECL	8.0~1200	4.2	57
NPG/SPE	EC	50~6000	8.36	58
ChOx/AuNPs/ssDNA-rGO/GCE	EC	7.5~280	2.1	59
ChOx/GA/bp-VMSF/ITO	ECL	50~5000	1.5	This work

β-CD, β-cyclodextrin; BQ, benzoquinone; Cys, cysteamine; N-GQDs, nitrogen doped graphene quantum dots; APTES, (3-aminopropyl) triethoxysilane; FTO, fluorine tin oxide; CPE, carbon paste electrode; MWCNT, multiwall carbon nanotubes; IL, ionic liquid; MO, microorganism; g-C_3N_4, graphitic carbon nitride; BPNSs, black phosphorus nanosheets; GCE, glassy carbon electrode; C-PDANTs, carbonized polydopamine nanotubes; ChOx, cholesterol oxidase; NPG, nanoporous gold; SPE, screen-printed electrode; AuNPs, gold nanoparticles; ssDNA, single-stranded DNA; rGO, reduced graphene oxide; GA, glutaraldehyde; bp-VMSF, bipolar and bilayer vertically-aligned mesoporous silica film; EC, electrochemical; ECL, electrochemiluminescence.
